# Long Term Sequelae from Childhood Pneumonia; Systematic Review and Meta-Analysis

**DOI:** 10.1371/journal.pone.0031239

**Published:** 2012-02-22

**Authors:** Karen Edmond, Susana Scott, Viola Korczak, Catherine Ward, Colin Sanderson, Evropi Theodoratou, Andrew Clark, Ulla Griffiths, Igor Rudan, Harry Campbell

**Affiliations:** 1 Department of Infectious Disease Epidemiology, London School of Hygiene and Tropical Medicine, London, United Kingdom; 2 Department of Global Health and Development, London School of Hygiene and Tropical Medicine, London, United Kingdom; 3 Department of Population Health Sciences, University of Edinburgh, Edinburgh, United Kingdom; 4 Department of Health Services Research and Policy, London School of Hygiene and Tropical Medicine, London, United Kingdom; Aga Khan University, Pakistan

## Abstract

**Background:**

The risks of long term sequelae from childhood pneumonia have not been systematically assessed. The aims of this study were to: (i) estimate the risks of respiratory sequelae after pneumonia in children under five years; (ii) estimate the distribution of the different types of respiratory sequelae; and (iii) compare sequelae risk by hospitalisation status and pathogen.

**Methods:**

We systematically reviewed published papers from 1970 to 2011. Standard global burden of disease categories (restrictive lung disease, obstructive lung disease, bronchiectasis) were labelled as major sequelae. ‘Minor’ sequelae (chronic bronchitis, asthma, other abnormal pulmonary function, other respiratory disease), and multiple impairments were also included. Thirteen papers were selected for inclusion. Synthesis was by random effects meta-analysis and meta-regression.

**Results:**

Risk of at least one major sequelae was 5.5% (95% confidence interval [95% CI] 2.8–8.3%) in non hospitalised children and 13.6% [6.2–21.1%]) in hospitalised children. Adenovirus pneumonia was associated with the highest sequelae risk (54.8% [39.2–70.5%]) but children hospitalised with no pathogen isolated also had high risk (17.6% [10.9–24.3%]). The most common type of major sequela was restrictive lung disease (5.4% [2.5–10.2%]) . Potential confounders such as loss to follow up and median age at infection were not associated with sequelae risk in the final models.

**Conclusions:**

All children with pneumonia diagnosed by a health professional should be considered at risk of long term sequelae. Evaluation of childhood pneumonia interventions should include potential impact on long term respiratory sequelae.

## Introduction

Pneumonia is the most common cause of mortality in children under five years of age. Almost 200 million new episodes of pneumonia occur each year in children under five years, 95% of them in developing countries. 10% are severe enough to be life-threatening and require hospital admission [1], [2].

It is well known that children with immunodeficiency and cystic fibrosis have long term respiratory problems such as recurrent pneumonia, bronchiectasis and restrictive lung disease [3]. Children and adults from marginalised and Indigenous populations also have recurrent pneumonia and frequent hospital admissions [4], [5]. Long term respiratory sequelae from pathogens such as Adenovirus and *Mycoplasma pneumoniae* have also been well documented including severe problems such as bronchiectasis and bronchiolitis obliterans, a respiratory disease in which the bronchioles are compressed and narrowed by inflammatory products and fibrosis [3], [6].

However, there is poor understanding of long term respiratory outcomes from pneumonia in children without risk factors or highly virulent pathogens; especially outcomes for children who have not been hospitalised. This information is needed to ensure that children with pneumonia are correctly managed by health professionals after they have recovered from their acute illness. These data are also needed to accurately assess the total long term burden of disease from childhood pneumonia and to understand the full impact of interventions against childhood pneumonia (e.g. vaccines against *Streptococcus pneumoniae* and reduction of indoor air pollution).

The aims of this study were to: (i) estimate the risks of respiratory sequelae after pneumonia in children under five years; (ii) estimate the distribution of the different types of respiratory sequelae; and (iii) compare sequelae risk by hospitalisation status and pathogen.

## Methods

This study was conducted using the PRISMA (Preferred reporting items for systematic reviews and meta-analyses) guidelines [7].

### Definitions

A case of ‘pneumonia’ was defined as any child under five years with clinical signs of pneumonia as diagnosed by a health professional (Table 1). Hospital pneumonia was defined as a child requiring hospitalisation for pneumonia. Non hospital pneumonia was defined as a child with no hospitalisation or whose hospitalisation status was unknown. Pathogen specific pneumonia was defined as any child under 5 years with clinical signs of pneumonia plus laboratory evidence (in blood or lung aspirate) of a recognised pneumonia pathogen e.g. *Streptococcus pneumoniae*, *Haemophilus influenzae* type b, *Staphlococcus aureus*, Adenovirus *Mycoplasma pneumoniae*, *Chlamydia pneumoniae*, or Respiratory syncitial virus. Non pathogen specific pneumonia was defined when laboratory tests were not performed or were inconclusive. Radiologically confirmed pneumonia was defined as radiological areas of opacity representing consolidation according to WHO criteria [8], [9].

**Table 1:** Pneumonia case definitions.

Hospital pneumonia	Any child under 5 years with clinical signs of pneumonia as diagnosed by a health professional that required hospitalisation
Non hospital pneumonia	Any child under 5 years with clinical signs of pneumonia as diagnosed by a health professional that did not require hospitalisation or hospitalisation status was unknown
Pathogen specific pneumonia	Any child under 5 years with clinical signs of pneumonia as diagnosed by a health professional with laboratory evidence (in blood or lung aspirate) of recognised pneumonia pathogen eg *Streptococcus pneumoniae*, *Haemophilus influenzae* type b, *Staphlococcus aureus*, *Mycoplasma pneumoniae*, Chlamydia *pneumonia*, Adenovirus, Respiratory syncitial virus
Non pathogen specific pneumonia	Any child under 5 years with clinical signs of pneumonia as diagnosed by a health professional where laboratory tests were not performed or were inconclusive

A ‘sequela’ was defined according to the 2001 global burden of disease (GBD) priorities project as a health state resulting from pneumonia for which epidemiological estimates (incidence, prevalence, average duration) and a single average disability weight could be calculated [10], [11]. It included all current and future functional health states (until remission to full health or death) in the natural history of the disease that impaired quality of life or activities of daily living. The 2001 GBD project defined a group of specific sequelae domains (Table 2) which we retained for use in this study (restrictive lung disease, obstructive lung disease, bronchiectasis) and labelled as ‘major’ sequelae [10]. We also collected data on other respiratory sequelae diagnosed by medical professionals but labelled these as ‘non GBD’ ‘minor’ sequelae (chronic bronchitis, asthma, other abnormal pulmonary function, other respiratory disease). A separate category was also created to identify individuals with more than one sequela (multiple impairments), as pneumonia can result in damage to a number of different loci within the respiratory system (eg bronchi and lung parenchyma) and deficits in many different domains. All sequelae were allocated an International Classification of Diseases Version 10 (ICD10) code [12].

**Table 2:** Sequelae domains and case definitions.

‘GBD’ ‘Major’ case definitions (ICD10 codes)	
Restrictive lung disease (J43)	Impaired lung function as measured by a reduced forced vital capacity (FVC) and a normal forced expiratory volume in 1 second (FEV1) to FVC ratio. Person has mild, moderate or severe breathing difficulties with or without wheeze
Obstructive lung disease (J44)	Impaired lung function as measured by a reduced FEV1 and a low FEV1 to FVC ratio. The impaired lung function does not improve significantly with bronchodilator therapy. Person has mild, moderate or severe breathing difficulties with wheeze which does not improve significantly with bronchodilator therapy
Bronchiectasis (J47)	Respiratory disease with localised, irreversible dilatation of part of the bronchial tree. Involved bronchi are dilated, inflamed, and easily collapsible, resulting in airflow obstruction and impaired clearance of secretions. Person has production of excessive amounts of sputum and frequent respiratory tract infections with or without wheeze
Multiple impairments	At least two of the above major domains

‘NonGBD’ ‘Minor’ case definitions (ICD10 codes)	
Chronic bronchitis (J41, J42, J44)	Chronic suppurative respiratory disease without bronchial dilatation or signs of bronchiectasis. Person has production of excessive amounts of sputum and frequent respiratory tract infections with or without wheeze.
Asthma (J45)	Impaired lung function as measured by a reduced FEV1 and a low FEV1 to FVC ratio. The impaired lung function does improve significantly with bronchodilator therapy. Person has mild, moderate or severe breathing difficulties with wheeze which does improve significantly with bronchodilator therapy.
Other abnormal pulmonary function (R94.2)	Impaired lung function as measured by FEV1 or FVC which does not satisfy criteria above.
Other chronic respiratory disease. (J98.9)	Other defined respiratory disease not classified above as diagnosed by a health professional using standard tools (eg questionnaires)
Multiple impairments	At least two of the above minor domains

GBD = Global Burden of Disease Project.

ICD10 = International Classification of Diseases, 10th edition.

FVC  =  Forced vital capacity (FVC).

FEV1  =  Forced expiratory volume in 1 second.

### Search strategy and selection criteria

**Figure 1:**
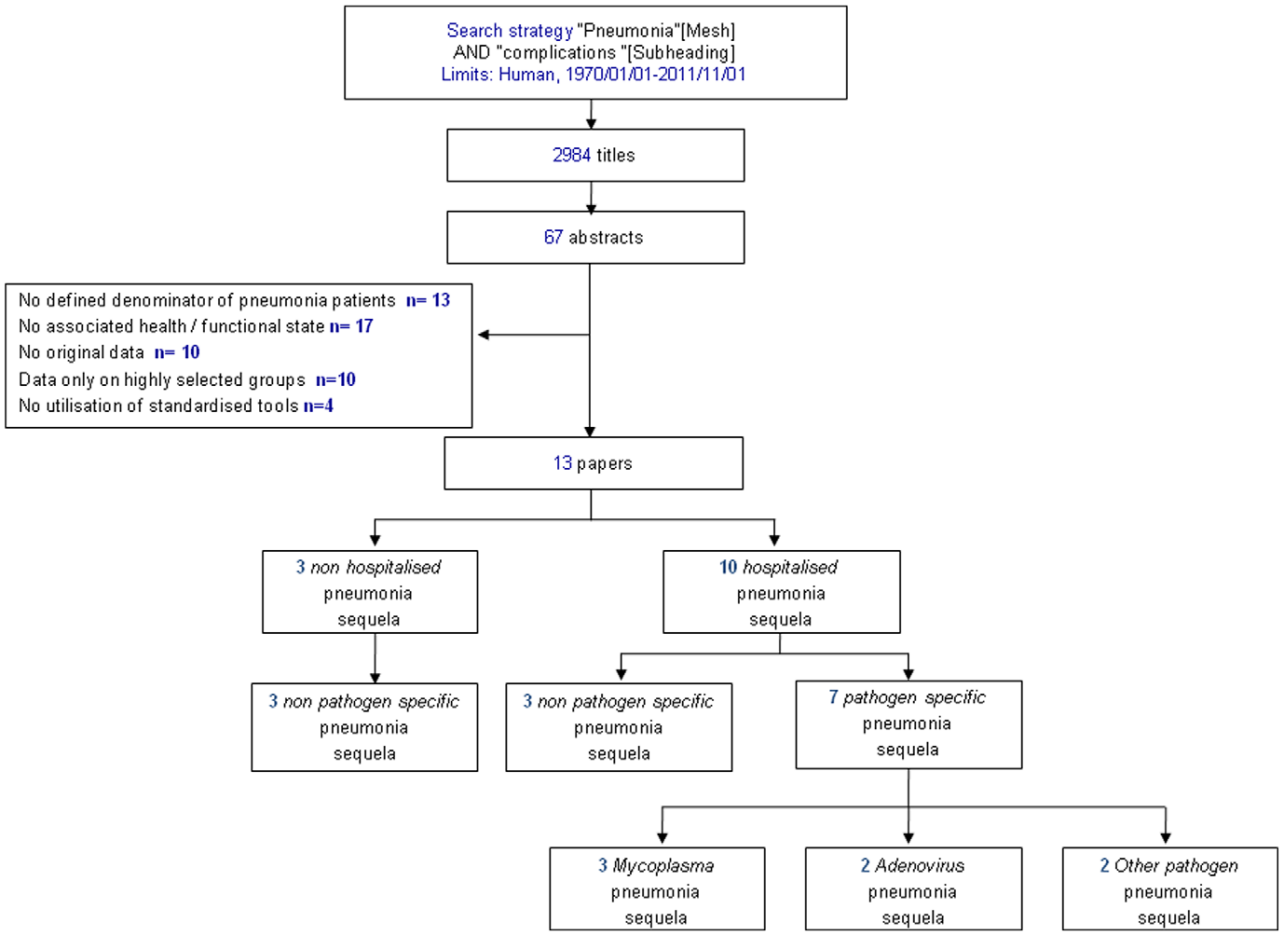
Search strategy.

The initial search aimed to be as inclusive as possible using search terms “Pneumonia” AND “complications” [Subheading] Limits: Human, 1970/01/01-2011/11/01 (Figure 1). We searched Medline, WHOLIS, EMBASE, CINAHL Plus, and Web of Science databases. We also reviewed reference lists of articles to identify additional papers and reports and contacted experts in the field to seek unpublished data and data that may have been missed by our search. There were no language restrictions.

Papers were excluded if they did not report on: a defined denominator of pneumonia patients (e.g. case series were excluded); sequelae of pneumonia (e.g. studies that only reported associated conditions such as septicaemia or arthritis were excluded); health/functional states; original data (e.g. reviews, repeated datasets were excluded); if they did not describe data representative of the whole population of children (e.g. studies containing only information on very high risk groups [e.g. cystic fibrosis, immunodeficiency] were excluded); and if the papers did not include any children who were under five years at the time of the pneumonia episode. Papers were also excluded if they did not examine for sequelae using standard tools (e.g. self reported conditions were excluded).

We also assessed the quality of the included studies with the Newcastle-Ottawa scales for assessing the quality of observational studies in meta-analyses [13]. We assigned risk of bias (low, moderate, and high risk of bias) as described by the Cochrane Handbook [14].

### Data extraction

Two reviewers examined titles, abstracts, and papers independently using identical case definitions, data abstraction forms and selection criteria. Disagreements were resolved by consensus between the two reviewers and the lead authors.

We collected basic data on author, study date, the number of children with pneumonia, the number of children who were followed up, duration of follow up, the final number who were examined for sequelae and classified the type of sequelae as major and minor according to the case definitions in Table 2. We also collected data on potential explanatory variables i.e. variables that may explain variance in risk of sequelae. For each study, categories for explanatory variables were created after an initial review of the data as follows: cause of pneumonia (pathogen specific vs non pathogen specific), hospitalisation status (hospitalised vs not hospitalised), study design (prospective vs retrospective), median age at infection (<2 years vs ≥2 years), gender (proportion male <50% vs ≥50%), duration of follow up (<2 years vs ≥2 years), proportion of individuals with pneumonia who were lost to follow up (<25% vs ≥25%). We also used data obtained from 2009 World Bank, World Health Organisation (WHO) and Child Health Epidemiology Reference Group (CHERG) data sets [15], [16], [17] to categorise countries into WHO region (African, South East Asian, Western Pacific, Eastern Mediterranean, European, and American regions), and GNI band in $US per capita from the year 2009 (low income, low middle income, high middle income, high income).

We also looked for data on other variables that are known to increase risk of respiratory sequelae such as birthweight, prematurity, pre-existing respiratory function (eg need for ventilatory support at birth), indoor air pollution, cigarette smoking, atopy and previous episodes of pneumonia but the classifications used were disparate and the data could not be synthesised. Where data were reported for multiple time periods, the most recent data were used.

### Analysis

Our initial review indicated that sequelae risk was heterogeneous and influenced by hospitalisation status and pathogenic cause. Thus we decided to present risks stratified into four groups (hospital pathogen specific, hospital nonpathogen specific, nonhospital pathogen specific, nonhospital nonpathogen specific) and to use random effects meta-analytic techniques to estimate the pooled risk of developing at least one major and minor sequela within these strata. We also used random effects meta analysis to calculate risks in each sequelae domain (restrictive lung disease, obstructive lung disease, bronchiectasis, chronic bronchitis, asthma, other disease and multiple disease).

Multi-level mixed-effects logistic regression was used to investigate the effect of hospitalisation status, pathogenic cause and other explanatory variables on pneumonia sequelae estimates. Pathogenic cause and hospitalisation status were included *apriori* in all models. Crude and adjusted odds ratios and their 95% CI were calculated. Statistical analyses were performed using STATA Release 12 statistical software (Stata, College Station, TX, USA).

## Results

### Search results

2984 papers published between 1^st^ January 1970 to 1^st^ November 2011 were identified from the Medline search and 67 titles were retained (Figure 1). No additional articles were obtained from the WHOLIS, EMBASE, CINAHL Plus and Web of Science searches, experts in the field and reference lists. No unpublished data that met our inclusion criteria were identified.

13 papers were retained after the abstracts were reviewed and our inclusion criteria were applied (Table 3). Three papers investigated sequelae risk in children who were not hospitalised [18], [19], [20], and none of these papers included pathogenic causes. Ten papers investigated sequelae risk in hospitalised children (3 nonpathogenic cause [21], [22], [23], 7 pathogenic cause [2 Adenovirus [24], [25], 3 *Mycoplasma pneumoniae* [26], [27], [28], 1 *Staphlococcus aureus* [29], 1 *Chlamydia pneumoniae* [30]) Radiological confirmation was available for all hospital studies but no non hospital studies.

**Table 3:** Characteristics of included studies, 1970–2011.

	Non hospital non pathogen	Hospital non pathogen	Hospital pathogen	Total
	3 papers	3 papers	7 papers	13 papers
WHO region				
Americas	0	0	2 (33%)	2 (15%)
Europe	3 (100%)	1 (33%)	2 (33%)	6 (46%)
Africa	0	2 (66%)	0	2 (15%)
Western Pacific	0	0	3 (43%)	3 (23%)
South East Asia	0	0	0	0
Eastern Mediterranean	0	0	0	0
GNI band (in $US per capita)				
High (≥12,196)	3 (100%)	1 (33%)	6 (86%)	10 (77%)
UMI (3,946–12,195)	0	1 (33%)	1 (14%)	2 (15%)
LMI (996–3945)	0	0	0	
Low (≤995)	0	1 (33%)	0	1 (8%)
Study design				
Prospective	3 (100%)	3 (100%)	0	6 (46%)
Retrospective	0	0	7 (100%)	7 (54%)
Median age at infection				
<2 years	3 (100%)	2 (66%)	1 (14%)	6 (46%)
≥2 years	0	1 (33%)	6 (86%)	7 (54%)
Duration of follow up				
<2 years	0	0	1 (14%)	1 (8%)
≥2 years	3 (100%)	3 (100%)	6 (86%)	12 (92%)
Loss to followup				
<25%	1 (33%)	1 (33%)	3 (43%)	5 (38%)
≥25%	2 (66%)	2 (66%)	4 (57%)	8 (62%)
Proportion male				
<50%	1 (33%)	1 (33%)	1 (14%)	3 (23%)
≥50%	2 (66%)	2 (66%)	6 (86%)	10 (77%)

Overall, 722 children with pneumonia were examined for sequelae (median number of children per study 38, interquartile range [IQR] 22–62) (Table 1) and 77 had major sequela. 439 (60.8%) children were under 2 years at the time of the pneumonia episode and none had risk factors. Characteristics of the 13 included studies are presented in Table 3 and full details can be found in Appendix S1.

Only one study had a duration of follow up of less than 2 years and the median duration of follow up was 10.8 years (IQR 2.1–17.0]. Five (38%) studies reported loss to followup rates under 25% and the median loss to follow up in all the studies was 34% (IQR 12–45%]). Six (46%) studies were prospective. 46% papers were from Europe, 23% from the Western Pacific region, and 15% were from the Americas and Africa. There were no papers from the Eastern Mediterranean or South East Asian regions. Ten (77%) papers were from high income countries and only one (5.9%) paper was from a low income country.

To assess whether any publication bias was likely in our paper we performed scatter plots of sequela risk against sample size and standard error but no correlation was found (p value = 0.305).

### Sequelae estimates

**Figure 2:**
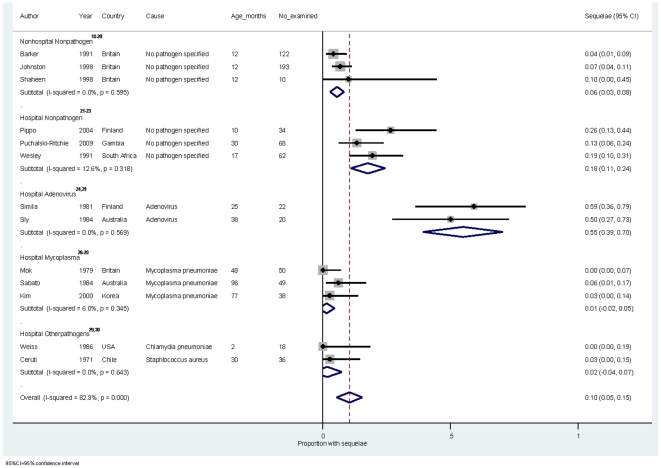
Meta-analysis of the risk of developing at least one major pneumonia sequela in children under five years by hospitalisation status and pathogenic cause, 1970–2011.

Risk of at least one major sequela was 5.5% (95% confidence interval [95% CI] 2.8–8.3%) in non hospitalised children (Table 4) and 13.6% [6.2–21.1%]) in hospitalised children. Figure 2 displays the risk of at least one major pneumonia sequela by hospital site and pathogenic cause. Risk appeared homogeneous within all strata; the *I* ^2^ (the percentage of variation attributable to heterogeneity) was below 15% for all analyses. Adenovirus pneumonia was associated with the highest sequelae risk (54.8% [39.2–70.5%]) but children hospitalised with no pathogen isolated also had high risk (17.6% [10.9–24.3%]). Risk of developing at least one minor sequela was 1.6% (1.0–8.5%) in non hospitalised children and 7.1% (1.0–13.4%) in hospitalised children (Table 4) .

**Table 4:** Major pneumonia sequelae risk, by site and pathogen, 1970–2011.

	Non hospital non pathogen specific	Hospital non pathogen specific	Hospital pathogen specific	Total
	Risk (95%CI)	Risk (95%CI)	Risk (95%CI)	Risk (95%CI)
	3 papers	3 papers	7 papers	13 papers
At least one major sequela	5.5% (2.8–8.3%)	17.6% (10.9–24.3%)	11.1% (2.5–19.7%)	10.4% (5.4–15.4%)
Restrictive lung disease	5.5% (3.0–8.0%)	17.1% (10.7–23.5%)	5.7% (2.2–8.6%)	5.4% (2.5–10.2%)
Obstructive lung disease	0	0	2.8% (0.05–6.4%)	2.8% (0.8–6.4%)
Bronchiectasis	0	0	0.9% (0.69–8.7%)	0.9% (0.7–8.7%)
Multiple impairments - Bronchiectasis combinedwith restrictive lung disease	0	0.5% (0.15–4.7%)	1.7% (0.87–4.9%)	1.2% (0.05–7.7%)
At least one minor sequela	1.6% (1.0–8.5%)	5.0% (3.3–8.5%)	7.5% (5.1–13.9%)	6.7% (2.2–11.2%)
Chronic bronchitis	1.6% (1.0–8.5%)	4.2% (2.6–8.1%)	2.8% (1.0–8.1%)	2.8% (1.8–8.1%)
Asthma	0	1.2% (1.3–9.5%)	0.9% (0.2–2.4%)	0.7% (0.2–4.2%)
Other abnormal pulmonary function	0	0	0	0
Other chronic respiratory disease	0	0	0	0
Multiple impairments - Chronic bronchitis combined with asthma	0	0	3.8% (1.6–5.1%)	3.2% (0.1–5.1%)
At least one major or minor sequela	7.1% (2.9–26.8%)	23.2% (13.3–32.8%)	18.6% (15.0–23.4%)	17.1 (7.6–6.6%)

95%CI = 95% confidence interval.

### Domains

All participants were examined for all major (restrictive lung disease, obstructive lung disease, bronchiectasis) and minor (chronic bronchitis, asthma, other abnormal pulmonary function, other respiratory disease) domains (Table 4). The most common type of major sequela was restrictive lung disease (5.5 [2.5–10.2%]). Bronchiectasis was only reported after hospitalised pneumonia (0.9% (0.7–8.7%) and obstructive lung disease was only reported after adenovirus pneumonia 2.8% (0.8–6.4%). The most common type of minor sequela was chronic bronchitis 2.8% (1.8–8.1%) followed by asthma 0.7% (0.2–4.2%). The only multiple sequelae syndromes reported were bronchiectasis combined with restrictive lung disease (1.2% [0.05–7.7%]) and chronic bronchitis combined with asthma (3.2% [0.1–5.1%]) (Table 4). All risks were higher in hospitalised than non hospitalised children.

### Explanatory factors

Table 2 shows the results from the crude and adjusted random effects logistic regression analysis of the effect of important factors (pathogenic cause, site, age at infection, duration of follow up, loss to follow up, gender, study design, WHO region, GNI band) on pneumonia sequelae risk in children under five years.

The only factor that appeared predictive of pneumonia risk in the univariable analysis was the proportion of children lost to follow up (<25% vs ≥25%) (Table 5). However, this did not remain significant in the multivariable analysis and there was no evidence of a trend in risk of sequelae when loss to follow up was examined as a continuous variable (odds ratio [OR] 0.036 [0.0005–2.45] p value = 0.123). Young children under two years of age had a higher risk of sequelae (13.4% (4.5–22.3%)) compared to older children 8.7% (3.1–14.3%) but this difference was not statistically significant (OR 0.82 [0.14–4.67]).

**Table 5:** Regression analyses of the effect of explanatory variables on major pneumonia sequelae risk in children under five years, 1970–2011.

	Studies (n = 13)	Participants		Subgroup estimates	Univariable regression	Multivariable regression
		Children examined (n)	Major sequelae cases (n)	Risk (95% CI)	Odds ratio (95% CI)	Adjusted odds ratio* (95% CI)
Cause						
Non pathogen specific	6 (%)	489	49	10.9% (5.4–0.16.4%)	1	1
Pathogen specific	7 (%)	233	28	11.1% (2.5–19.7%)	1.60 (0.27–9.59)	3.33 (0.62–18.1)
					P value = 0.605	P value = 0.608
Hospitalisation status						
Non hospitalised	3 (%)	325	19	5.5% (2.8–8.3%)	1	1
Hospitalised	10 (%)	397	58	13.6% (6.2–21.1%)	1.93 (0.26–14.2)	3.65 (1.96–6.80)
					P value = 0.517	P value = 0.003
Median age at infection						
2 years	7 (%)	439	40	13.4% (4.5–22.3%)	1	-
≥2 years	6 (%)	283	37	8.7% (3.1–14.3%)	0.82 (0.14–4.67)	-
					P value = 0.824	-
Proportion male						
<50%	3 (%)	279	22	6.8% (1.0–12.6%)	1	-
≥50%	10 (%)	443	55	13.0% (6.2–19.7%)	1.97 (0.25–15.4)	-
					P value = 0.516	-
Duration of follow up						
<2 years	1 (%)	38	1	2.6% (−4.2–9.5%)	1	-
≥2 years	12 (%)	684	76	11.5% (6.0–16.9%)	5.51 (0.16–192.83)	-
					P value = 0.347	-
Loss to follow up						
<25%	5 (%)	234	38	6.1% (1.8–10.4%)	1	1
≥25%	8 (%)	488	39	24.8% (0.92–40.4%)	0.22 (0.048–1.05)	0.93 (0.47–1.81)
					P value = 0.058	P value = 0.821
Study design						
Prospective	7 (%)	489	49	10.9% (5.4–16.4%)	1	-
Retrospective	6 (%)	233	28	11.1% (2.5–19.7%)	0.62 (0.10–3.73)	-
					P value 0.605	-
WHO region						
Americas	2 (%)	54	1	1.7% (−4.0–7.4%)	1	-
Europe	6 (%)	431	41	11.6% (3.9–19.2%)	10.4 (0.45–240.0)	-
Africa	2 (%)	130	21	15.7% (9.0–22.4%)	17.4 (0.54–561.4)	-
Western Pacific	3 (%)	107	14	14.8% (−1.4–30.9%)	11.9 (0.42–337.5)	-
South East Asia	0	-	-	-	-	-
Eastern Mediterranean	0	-	-	-	-	-
					P value = 0.286	-
GNI band (in $US per capita)						
High (≥12,196)	10 (%)	556	55	10.2% (4.4–16.1%)	1	-
UMI (3,946–12,195)	2 (%)	98	13	10.6% (−5.6–26.8%)	0.91 (0.08–10.2)	-
LMI (996–3945)	0	-	-	-	-	-
Low (≤995)	4 (%)	68	9	13.2% (4.5–21.9%)	1.56 (0.07–34.1)	-
					P value = 0.811	-

*Adjusted for pathogenic cause of pneumonia, hospitalisation status, loss to followup.

The final multivariable model adjusted for the effects of pathogenic cause, hospitalisation status and loss to follow up. In this model only hospitalisation status remained strongly associated with major pneumonia sequelae risk; there was a 3 fold greater risk of sequelae in children hospitalised with pneumonia than non hospitalised children (adjusted OR 3.65 [1.96–6.80]).

## Discussion

We reported an overall risk of long term major respiratory sequelae from childhood pneumonia in non hospitalised children of 5.5% . Risk was three times higher in hospitalised children. Children hospitalised with no pathogen isolated had a 20% increase in risk. More than half of the children hospitalised with adenovirus pneumonia were reported to have respiratory sequelae.

Our findings are consistent with large population based studies of children born in the early 1900 s [18], [19], [20], [31], [32], [33]. These studies reported a 6–7% increased risk of restrictive lung disease in mid adulthood from childhood pneumonia. These studies were of high quality with careful data collection, assessment of pulmonary function and ascertainment of sequelae. It has also been suggested that malnourished children and poor children who live in over crowded households and may have an even greater increased risk of long term sequelae [3], [23], [34], [35]. However, we were unable to assess risk in low income settings as we included only one study from a low income country. There were also only two studies from Africa and none from Asia.

Our high risks in hospital patients are also consistent with other studies [36], [37], [38]. Hospitalised children are more likely to have severe sequelae than children who are treated at home [39]. Children who are hospitalised for pneumonia have severe disease and require oxygen, intravenous antibiotics and some need ventilatory support [36] [37]. They have longer duration of illness, greater damage to lung parenchyma and bronchioles and are more likely to develop nosocomial pneumonia infections with virulent and antibiotic resistant organisms such as *Pseudomonas* and *Klebsiella spp* [40], [41].

We reported that young children under two years of age had a higher risk of sequelae compared to older children but this difference was not statistically significant. Other studies have indicated that infants under 12 months have the highest risk of pneumonia sequelae [3], [19], [20]. Indeed, it has been suggested that lower respiratory tract infection at an early stage of lung development may impair lung growth and reduce subsequent lung function [3]. This could be due to harmful effects of the disease on growing lungs or halted lung growth because of injury [42].

Restrictive lung disease was the most common type of sequela. This is in keeping with other studies which indicate that pneumonia pathogens can damage interstitial lung parenchyma causing acute lung consolidation [3], [31]. Expansion and growth of alveoli can be reduced resulting in reduced vital capacity and proportional reduction in forced expiratory volume [19], [43], [44]. Pneumonia pathogens also can damage bronchiolar epithelium and connective tissue resulting in bronchiolar dilatation, impaired clearance of secretions and chronic suppurative lung disease including bronchiectasis [43], [44]. The link between childhood pneumonia and obstructive lung disease is less clear. We reported a low risk of obstructive lung disease and only after Adenovirus infection. Other studies have also reported low risk of obstructive lung disease after childhood pneumonia [6], [19], [20], [31]. Interestingly, increased risk of obstructive lung disease has been reported in infants with intrauterine malnutrition born low birth weight compared to normal birth weight infants [19], [20]. This has been attributed to impairment of airway growth and abnormally narrow airway size at birth. However, we did report an increased risk of asthma in hospitalised children with and without pathogen specific pneumonia which requires more investigation.

Children who have poor respiratory function at birth are at risk of developing both pneumonia and later long term respiratory problems [3], [45]. A signficant limitation of our meta-analysis and other studies to date is the lack of information on baseline respiratory status prior to the pneumonia episode. These baseline data were not recorded in any of our included studies. However, other studies have used birth weight as a proxy for early respiratory function and investigated effects of birth weight on pneumonia and long term respiratory outcomes [19], [20]. These studies demonstrated important associations between childhood pneumonia and long term outcome even after adjusting for birth weight. Other important confounders include cigarette smoking and indoor airpollution [32], [46], but we were also unable to control for these factors in our multivariable analyses. We had no information on human immunodeficiency virus (HIV) infection in our study populations, and only two studies were conducted in countries where HIV infection is highly prevalent [21], [23].

There were other limitations to our meta-analysis. Firstly, our median loss to follow up was 34% and it is likely that the loss to follow up was differential; i.e. families with unwell children were more likely to present for follow up than those with well children. However, our studies had long follow up time (median 11 years) so we were able to assess outcomes also in adulthood which is an important time for ascertainment of respiratory sequelae [18], [45]. Secondly, other disease syndromes such as septicaemia, dehydration, acidosis and malaria can be misclassified as pneumonia [1], [3]. Radiologically confirmed consolidation is the gold standard for diagnosis of pneumonia but radiological confirmation was only available in the hospital studies. However, we used strict pneumonia case definitions which required a pneumonia diagnosis by a health professional. We also used GBD and ICD10 definitions to categorise our outcomes clearly and were able to stratify sequelae into major, minor, specific domains and multiple impairments. Finally, our included studies were heterogeneous and could only be analysed in specific subgroups. Also, many had small sample sizes. More long term follow up studies are needed, especially population based birth cohort studies from low income countries and studies with respiratory function ascertained soon after birth.

Our study has a number of implications for program and policy development. All children with pneumonia diagnosed by a health professional should be considered to be at risk of long term sequelae; even if the pneumonia episode is only a marker of pre-existing respiratory dysfunction. Currently, most paediatricians regard childhood pneumonia to be an acute disease with no need for long term follow up if a child has no risk factors and a clear chest radiograph. We consider that all children under five years who have a diagnosis of pneumonia should have at least one follow up appointment with a health professional after the acute symptoms have resolved. Evaluation of childhood pneumonia interventions should also include potential impact on long term respiratory sequelae.

## Supporting Information

Appendix S1 **Details and quality of included studies.**
(XLSX)
